# Sex difference in Double Iron ultra-triathlon performance

**DOI:** 10.1186/2046-7648-2-12

**Published:** 2013-04-01

**Authors:** Katrin Sigg, Beat Knechtle, Christoph A Rüst, Patrizia Knechtle, Romuald Lepers, Thomas Rosemann

**Affiliations:** 1 Institute of General Practice and Health Services Research, University of Zurich, Zurich, Switzerland; 2 Gesundheitszentrum St. Gallen, St. Gallen, Switzerland; 3 INSERM U1093, Faculty of Sport Sciences, University of Burgundy, Dijon, France; 4 Facharzt FMH für Allgemeinmedizin, Gesundheitszentrum St. Gallen, Vadianstrasse 26, St. Gallen, 9011, Switzerland

**Keywords:** Triathlon, Ultra-endurance, Swimming, Cycling, Running

## Abstract

**Background:**

The present study examined the sex difference in swimming (7.8 km), cycling (360 km), running (84 km), and overall race times for Double Iron ultra-triathletes.

**Methods:**

Sex differences in split times and overall race times of 1,591 men and 155 women finishing a Double Iron ultra-triathlon between 1985 and 2012 were analyzed.

**Results:**

The annual number of finishes increased linearly for women and exponentially for men. Men achieved race times of 1,716 ± 243 min compared to 1,834 ± 261 min for women and were 118 ± 18 min (6.9%) faster (*p* < 0.01). Men finished swimming within 156 ± 63 min compared to women with 163 ± 31 min and were 8 ± 32 min (5.1 ± 5.0%) faster (*p* < 0.01). For cycling, men (852 ± 196 min) were 71 ± 70 min (8.3 ± 3.5%) faster than women (923 ± 126 min) (*p* < 0.01). Men completed the run split within 710 ± 145 min compared to 739 ± 150 min for women and were 30 ± 5 min (4.2 ± 3.4%) faster (*p* = 0.03). The annual three fastest men improved race time from 1,650 ± 114 min in 1985 to 1,339 ± 33 min in 2012 (*p* < 0.01). Overall race time for women remained unchanged at 1,593 ± 173 min with an unchanged sex difference of 27.1 ± 8.6%. In swimming, the split times for the annual three fastest women (148 ± 14 min) and men (127 ± 20 min) remained unchanged with an unchanged sex difference of 26.8 ± 13.5%. In cycling, the annual three fastest men improved the split time from 826 ± 60 min to 666 ± 18 min (*p* = 0.02). For women, the split time in cycling remained unchanged at 844 ± 54 min with an unchanged sex difference of 25.2 ± 7.3%. In running, the annual fastest three men improved split times from 649 ± 77 min to 532 ± 16 min (*p* < 0.01). For women, however, the split times remained unchanged at 657 ± 70 min with a stable sex difference of 32.4 ± 12.5%.

**Conclusions:**

To summarize, the present findings showed that men were faster than women in Double Iron ultra-triathlon, men improved overall race times, cycling and running split times, and the sex difference remained unchanged across years for overall race time and split times. The sex differences for overall race times and split times were higher than reported for Ironman triathlon.

## Background

Triathlon is a multi-sports competition covering the three endurance disciplines swimming, cycling, and running in this subsequent order. The most popular long-distance triathlon is the Ironman distance triathlon covering 3.8 km of swimming, 180 km of cycling, and 42 of km running [1-3]. The first Ironman triathlon was held in 1978 in Honolulu, Hawaii, and involved 12 male finishers [1]. Apart from the Ironman distance triathlon, longer triathlon distances do exist from the Double Iron ultra-triathlon covering 7.6 of km swimming, 360 of km cycling, and 84.4 of km running [4], up to the Double Deca Iron ultra-triathlon covering 78 km of swimming, 3,600 km of cycling, and 844 km of running [5,6]. The first Double Iron ultra-triathlon was held in 1985 in Huntsville (USA), where 23 men finished [4]. Four years later, the first European Double Iron ultra-triathlon was held in Colmar (France) [7]. Since the first event in 1985, participation progressively increased with an improvement of race times [4].

The sex difference in endurance performance has been investigated for different disciplines and distances. Several studies investigating sex differences focused on running [8-13]. In marathon running, the best men achieved ~11% faster running times than the best women [13]. For other distances, a comparison of the world best running times from 100 m to 200 km showed that longer distances were associated with greater sex differences, with men being ~12.4% faster than women [9]. Similar findings were reported for shorter distances where men were, on average, ~11% faster than women in a 1,500-m run [13]. In long-distance swimming, women seemed to be able to achieve similar performances like men [14,15]. However, when peak swim performance for the annual fastest ultra-distance swimmers was analyzed, a sex difference of ~11%–12% resulted over time [16,17].

In cycling, the results of the World Track Cycling Championships from 1979 to 1999 in 200- and 1,000-m individual and team pursuit races for elite and junior athletes were investigated. The sex difference was ~11 ± 1.8% in all disciplines at all ages [18]. Regarding triathlon, the sex differences in race times differ between the disciplines in ‘Ironman Hawaii’. Lepers [1] reported that the sex differences in Ironman Hawaii from 1988 to 2007 were smaller for swimming (~9.8%) than for cycling (~12.7%) and running (~13.3%), with a sex difference of ~12.6% for overall race time. This finding was confirmed by analyzing the top ten men and women triathletes of the best age groups in Ironman Hawaii from 2006 to 2008 [2]. Lepers and Maffiuletti [2] reported a smaller sex difference in swimming (~12.1%) than in running (~18.2%) or cycling (~15.4%).

Regarding the change in sex difference over time, previous studies suggested a decrease in sex differences and a stabilization afterwards [13]. In marathon running, the sex difference in performance between women and men has stabilized since 1988 at ~12%. In the early 1980s, the sex difference was narrowing from ~11.4% in 1980 to ~9.7% in 1983 [13]. In Ironman Hawaii, performances in swimming, cycling, running, and overall race times showed no changes during the last two decades, neither for men nor for women [1]. In swimming, cycling, and overall race times, the changes were between ~1.1% and ~0.02% per decade [1]. The running performance changed by ~1.4% per decade. Since 1988, women improved their running performance by ~3.8% per decade [1]. When the changes in sex difference in Ironman Hawaii were investigated between years 1983 and 2012, the sex difference in overall race time decreased from ~15.2% to ~11.3% [3]. For the split disciplines, the sex difference remained unchanged for swimming (~12.5%) and cycling (~12.5%) but decreased for running from ~13.5% to ~7.3% [3]. For longer triathlon distances such as the Triple Iron ultra-triathlon covering 11.4 km of swimming, 540 km of cycling, and 126.6 km of running, the sex difference in overall performance increased from ~10% in 1992 to ~42% in 2011 for the annual fastest finishers [19]. For the annual fastest swimmers, the sex difference remained unchanged. In cycling and running, however, the sex differences increased from ~12% to ~40% and from ~10% to ~64%, respectively.

To date, no study investigated the changes in sex difference across years for the Double Iron ultra-triathlon distance. The present study intended to fill a gap in literature by investigating the changes in sex difference in Double Iron ultra-triathlon race times from the very first event in 1985 until 2012. Based upon present literature for Ironman Hawaii and Triple Iron ultra-triathlon, we hypothesized an unchanged sex difference in the swim performance but a decrease in the sex difference in performance in Double Iron ultra-triathlons across years.

## Methods

The data set from this study was obtained from the race website of the International Ultra-Triathlon Association (IUTA) [20] and from the Race Directors. The split times in swimming, cycling, running, and overall race times of all finishers in Double Iron ultra-triathlons held all over the world between 1985 and 2012 were analyzed. This study was approved by the institutional review board of St. Gallen, Switzerland, with a waiver for the requirement of an informed consent, given that the study involved the analysis of publicly available data.

### Data analysis

In total, data were available from 2,207 athletes, including 202 women (9.2%) and 2,005 men (90.8%). These 2,207 athletes participated in 29 races held in 17 different countries, leading to a number of 104 investigated events. Among the 2,207 athletes, 408 men (18.5%) and 47 women (2.1%) did not finish the race. Additionally, in 2007 and 2011, one male athlete was disqualified from each. Finally, data from 1,752 finishers (79.4% of all starters) including 1,597 men (91.2%) and 155 women (8.8%) could be analyzed. For the analysis of the development of race time in sex and sex difference, the split times per discipline (i.e., swimming, cycling, and running) and overall race time from the annual fastest and annual three fastest women and men were analyzed. In case the number of female and male finishers was below the required amount in a year, the respective year was excluded from data analysis.

### Statistical analysis

In order to increase the reliability of data analyses, each set of data was tested for normal distribution as well as for homogeneity of variances prior to statistical analyses. Normal distribution was tested using a D’Agostino and Pearson omnibus normality test, and homogeneity of variances was tested using Levene’s test. To find significant changes in the development of a variable across years, regression analyses were used. To find significant differences between two groups, Student’s *t* test was used in case of normal distributed data (with Welch’s correction in case of heteroscedasticity), and a Mann–Whitney test was used in case of not normal distributed data. Statistical analyses were performed using IBM SPSS Statistics (Version 19, IBM SPSS, Chicago, IL, USA) and GraphPad Prism (Version 5, GraphPad Software, La Jolla, CA, USA). Data in the text are given as mean and standard deviation. Sex difference was calculated using the formula: (race time [women] − race time [men]) / race time [men] × 100. The sex difference was calculated for every pairing of equally placed athletes (e.g., between a woman and man at first place, between a woman and man at second place, etc.) before calculating mean value and standard deviation of all the pairings. In order to facilitate reading, all sex differences were transformed to absolute values before analysis. For all statistical tests, significance was accepted at *p <* 0.05 (two-sided for *t* tests).

## Results

### Participation trends

**Figure 1:**
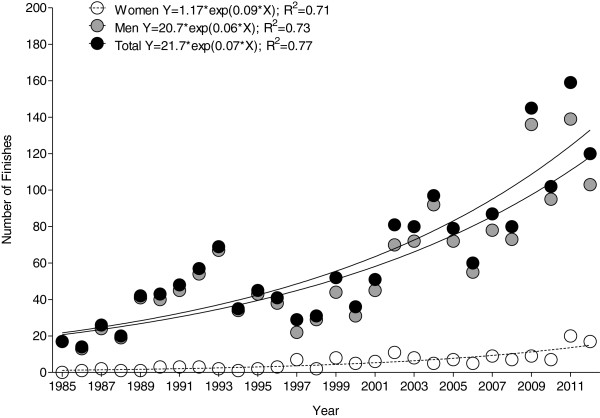
**Annual number of female, male, and overall finishes.** Formulas refer to relative year of analysis (1985 = year 0; 2012 = year 27).

Between 1985 and 2012, a total of 1,591 men (88.8%) and 155 women (78.3%) finished successfully a Double Iron ultra-triathlon, leading to a mean annual number of 4 ± 5 female finishes and 52 ± 34 male finishes. The annual number of finishes increased linearly for women but exponentially for men (Figure 1). The first woman started in 1985, most women finished in 2011 with 20 female finishers, and the highest percentage of female finishers was recorded for 1997 with 24.1%. On average, female participation accounted for 8.4% of the annual field.

### Sex difference in overall race times

**Figure 2:**
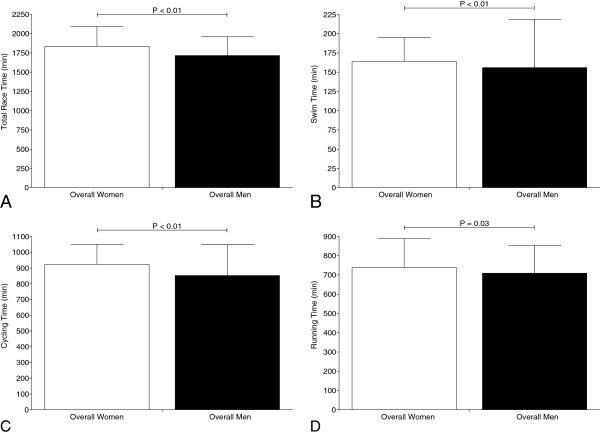
Overall race times (A), split times in swimming (B), cycling (C), and running (D) of all female and male finishers ever.

The 1,591 male finishers achieved a mean overall race time of 1,716 ± 243 min compared to the 155 female finishers with 1,834 ± 261 min and were 118 ± 18 min (6.9%) faster (*p* < 0.01) (Figure 2A). In swimming, overall men finished within 156 ± 63 min compared to overall women with 163 ± 31 min and were 8 ± 32 min (5.1 ± 5.0%) faster (*p* < 0.01) (Figure 2B). For cycling, overall men (852 ± 196 min) were 71 ± 70 min (8.3±3.5%) faster than overall women (923 ± 126 min) (*p* < 0.01) (Figure 2C). Overall men completed the run split within 710 ± 145 min compared to 739 ± 150 min for women and were 30 ± 5 min (4.2 ± 3.4%) faster (*p* = 0.03) (Figure 2D).

**Figure 3:**
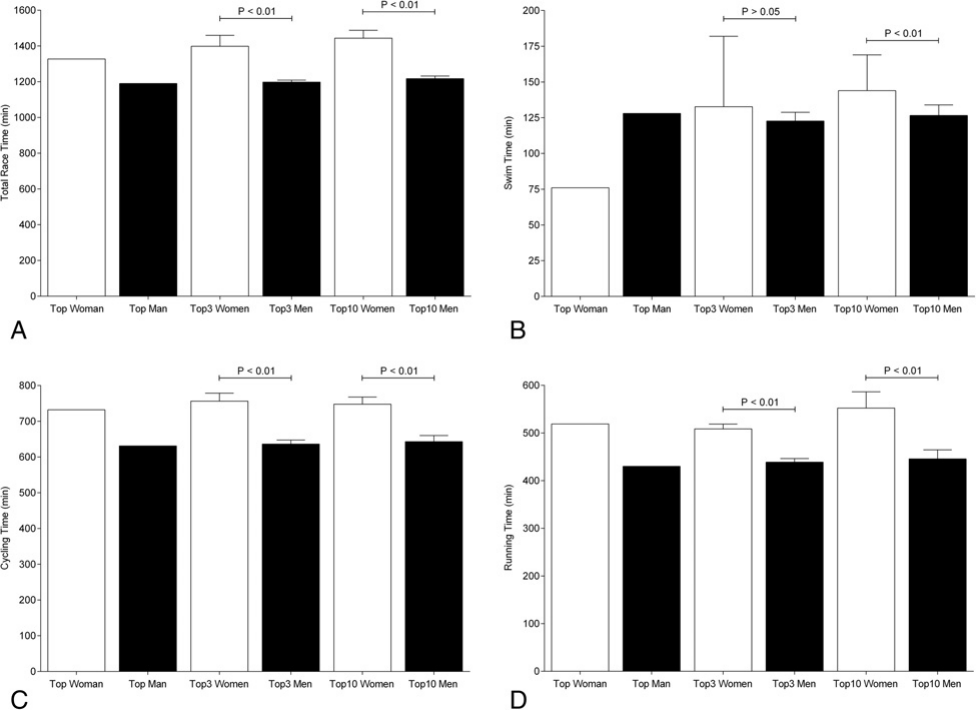
Overall race times (A), split times in swimming (B), cycling (C), and running (D) of the fastest, the three fastest, and the ten fastest women and men ever.

Figure 3 presents the overall race times and the split times of the fastest ever, the three fastest ever, and the ten fastest ever athletes. The actual world record in Double Iron ultra-triathlon for women was set in 1994 by Tina Bischoff (USA) in 22.11 h in Huntsville (USA). For men, Adrian Brennwald (Switzerland) achieved the fastest overall race time with 19.83 h in Neulengbach (Austria) in 2011. The fastest man ever was 137 min (11.5%) faster for overall race time (Figure 3A). Regarding the split times, the fastest woman ever achieved 76 min in swimming and was 52 min (40%) faster than the fastest man ever with 128 min (Figure 3B). In cycling, the fastest man ever finished after 631 min and was 101 min (16%) faster than the fastest woman ever (Figure 3C). In running, the fastest man ever was 89 min (20.7%) faster with 430 min compared to 519 min for the fastest woman ever (Figure 3D).

The three fastest women ever achieved 1,397 ± 61.7 min and were 199.0 ± 50.5 min (16.6 ± 4.5%) slower than the three fastest men ever with 1,198 ± 11.1 min (*p* < 0.01) (Figure 3A). In swimming, the three fastest women ever (132.7 ± 49.2 min) were not slower compared to the three fastest men (122.7 ± 6.1 min) (*p* > 0.05) (Figure 3B). In cycling, the three fastest men ever finished after 636.0 ± 11.4 min compared to the three fastest women ever with 756.3 ± 33.4 min and were 120 ± 11 min (18.9 ± 9.6%) faster (*p* < 0.01) (Figure 3C). In running, the three fastest men ever finished within 438.7 ± 7.8 min and were 70.0 ± 2.2 min (15.9 ± 2.9%) faster (*p* < 0.01) than the three fastest women ever with 508.7 ± 10.0 min (Figure 3D).

The ten fastest women ever achieved 1,444 ± 43.9 min and were 227 ± 0 min (18.6 ± 2.0%) slower than the ten fastest men ever with 1,217 ± 14.5 min (*p* < 0.01) (Figure 3A). In swimming, the ten fastest women ever (143.9 ± 25.0 min) were 17.3 ± 17.6 min (13.7 ± 2.3%) slower compared to the ten fastest men ever (126.6 ± 7.4 min) (*p* < 0.01) (Figure 3B). In cycling, the ten fastest men ever finished within 643.0 ± 17.2 min compared to the ten fastest women ever with 747.7 ± 20.1 min and were 104.7 ± 2.9 min (16.3 ± 16.8%) faster (*p* < 0.01) (Figure 3C). The ten fastest men ever completed the run within 445.7 ± 19.0 min and were 106.6 ± 15.2 min (23.9 ± 8.0%) faster (*p* < 0.01) compared to the ten fastest women ever with 552.3 ± 34.2 min (Figure 3D).

### Sex difference in the annual fastest race times

**Figure 4:**
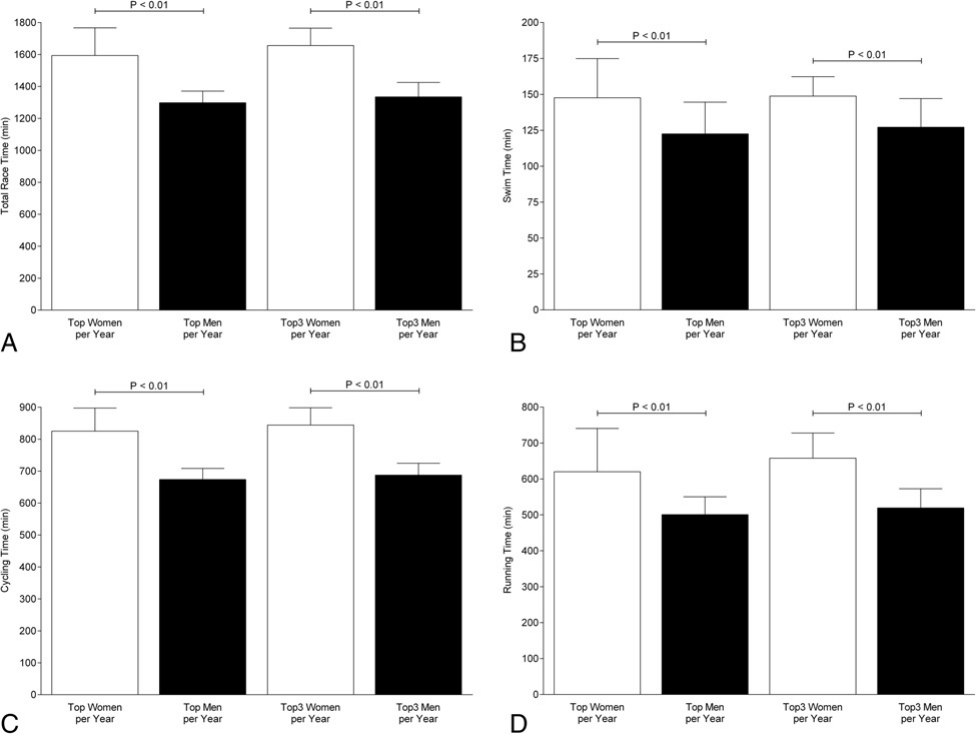
Overall race times (A), split times in swimming (B), cycling (C), and running (D) of the annual fastest and the annual three fastest women and men.

Figure 4 presents the overall race times (panel A), swim split times (panel B), cycling split times (panel C), and running split times (panel D) for the annual fastest and the three annual fastest women and men. For overall race times, the annual fastest men achieved 1,298 ± 73 min compared to women with 1,594 ± 173 min and were 304 ± 179 min (23.7±13.1%) faster (*p* < 0.01) (Figure 4A). In swimming, the annual fastest men achieved 122 ± 22 min and were 25 ± 27 min (24.8 ± 35.6%) faster than the annual fastest women with 147 ± 27 min (*p* < 0.01) (Figure 4B). In the cycling split, the annual fastest men (673 ± 34 min) were 154 ± 80 min (23.4 ± 12.6%) faster than the annual fastest women (825 ± 72 min) (*p* < 0.01) (Figure 4C). In running, the annual fastest men achieved 500 ± 49 min compared to the annual fastest women with 620 ± 120 min and were 124 ± 100 min (23.3 ± 20.8%) faster (*p* < 0.01) (Figure 4D).

Regarding the annual three fastest athletes, men achieved 1,335 ± 90.2 min for overall race times compared to women with 1,656 ± 109 min and were 321 ± 19 min (27.0 ± 10.7%) faster (*p* < 0.01) (Figure 4A). In swimming, the annual three fastest men achieved 127 ± 20 min and were 21.7 ± 6.6 min (26.7 ± 21.0%) faster than the annual three fastest women with 149 ± 13 min (*p* < 0.01) (Figure 4B). In the cycling split, the annual three fastest men (687 ± 34 min) were 157 ± 17 min (25.1 ± 11.8%) faster than the annual three fastest women (844 ± 54 min) (*p* < 0.01) (Figure 4C). In running, the annual three fastest men achieved 519 ± 53 min compared to the annual three fastest women with 657 ± 69 min and were 138 ± 16 min (32.4 ± 19.9%) faster (*p* < 0.01) (Figure 4D).

### Changes in sex difference over time

**Figure 5:**
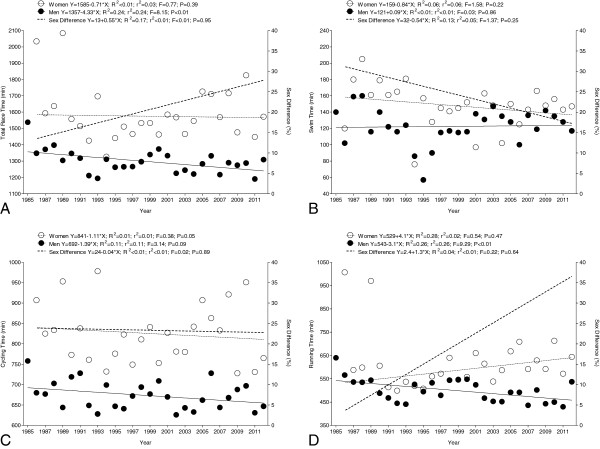
**Overall race times (A), split times in swimming (B), cycling (C), and running (D) of the annual fastest women and men.** Formulas refer to the relative year of analysis (1985 = year 0; 2012 = year 27).

The annual fastest men improved their overall race time from 1,538 min in 1985 to 1,309 min in 2012 (Figure 5A). Overall race time for the annual fastest women remained, however, unchanged at 1,593 ± 173 min with a stable sex difference of 23.7 ± 13.1% across years. In swimming, the split times for the annual fastest women (147 ± 27 min) and men (122 ± 22 min) remained unchanged with an unchanged sex difference of 29.7 ± 31.5% (Figure 5B). Also in cycling, the split times for the annual fastest women (825 ± 72 min) and men (673 ± 34 min) remained unchanged with an unchanged sex difference of 23.4 ± 12.6% (Figure 5C). In running, however, the annual fastest men improved their split time from 640 min in 1985 to 537 min in 2012 (Figure 5D). The split remained, however, unchanged for the annual fastest women at 620 ± 120 min with a stable sex difference of 25.4 ± 20.7%.

**Figure 6:**
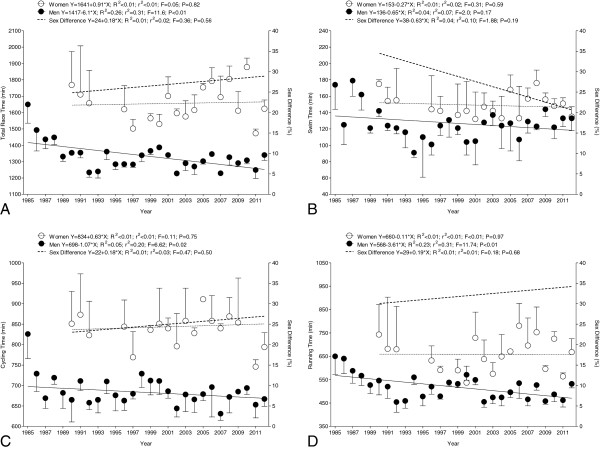
**Overall race times (A), split times in swimming (B), cycling (C), and running (D) of the annual three fastest women and men.** Formulas refer to the relative year of analysis (1985 = year 0; 2012 = year 27).

Regarding the annual three fastest athletes, men improved their overall race time from 1,650 ± 114 min in 1985 to 1,339 ± 33 min in 2012 (Figure 6A). Overall race time for the annual three fastest women remained unchanged at 1,593 ± 173 min with a stable sex difference of 27.1 ± 8.6% across years. In swimming, the split times for the annual three fastest women (148 ± 14 min) and men (127 ± 20 min) remained unchanged with a stable sex difference of 26.8 ± 13.5% (Figure 6B). In cycling, however, the annual three fastest men improved the bike split time from 826 ± 60 min in 1985 to 666 ± 18 min in 2012. For women, the split time in cycling remained unchanged for the annual three fastest finishers at 844 ± 54 min with a stable sex difference of 25.2 ± 7.3% (Figure 6C). Also, in running, the annual fastest three men improved the split times from 649 ± 77 min in 1985 to 532 ± 16 min in 2012. For women, however, the split times in running remained unchanged at 657 ± 70 min with a stable sex difference of 32.4 ± 12.5% (Figure 6D).

## Discussion

The aim of this study was to investigate the sex difference in the Double Iron ultra-triathlon races held between 1985 and 2012. The main findings were the following: (1) men were faster than women for overall race times and split times, (2) men improved in the overall race times and split times in cycling and running, (3) the sex difference remained unchanged over time for both overall race time and split times, and (4) the sex differences for overall race times and split times were higher than reported for Ironman triathlon.

### Participation trends

The number of male finishes increased exponentially over time, whereas the number of female finishes increased only linearly. Overall, women accounted for 8.4% of the field. The number of female finishes in Double Iron ultra-triathlons increased from 2% in the late 1980s up to 10% since 2001. In other ultra-distances such as running, an increase in women’s participation has been reported since 1977 [10,11]. In the ‘Western States 100-Mile Endurance Run’ in the USA, the percentage of female athletes increased from 10% to 20% in the late 1980s to 20% to 22% since 2001 [1]. The reason for this increase might be the higher number of participants aged 40 years and older [11], the higher number of women participating [10], and the increasing number of finished races per participant [11]. These changes in female participation might be explained by changes in society. In the last years, the daily life of a woman came closer to men’s life concerning work, family and hobbies, claims, desires, and possibilities. Krouse et al. [21] investigated the motivation of female ultra-marathoners competing in running events exceeding the 26.2-mile distance. The majority of these women were full-time workers, married, childless, task oriented, and therefore not having the characteristics of women some decades ago [21].

The number of male finishes was, however, considerably higher and increased exponentially. Physically active men seemed to have a different motivation to compete in races compared to women. Krouse et al. [21] described female ultra-runners as task oriented, internally motivated, and health and financially conscious individuals. Men, however, seemed to prefer sports requiring skills needed for success in male–male physical competition [22]. Additionally, men seemed to have a greater training motivation compared to women [23,24]. These characteristics might explain both the exponential increase in male finishes and the low female participation.

### Sex difference in performances

Men were faster than women, and men improved in overall race times, cycling, and running split times. The cycling and running split times were most probably responsible for the sex difference. The sex differences in Double Iron ultra-triathlon in overall race time and split times were greater than reported for shorter triathlon distances such as the Ironman triathlon. Lepers [1] reported sex differences of 9.8% in swimming, 12.7% in cycling, 13.3% in running, and 12.6% in overall race time in the Ironman Hawaii when investigating the time period 1981–2007. For the time period 2006–2008, Lepers and Maffiuletti [2] reported a lower sex difference in swimming (12.1%) than in cycling (15.4%) and running (18.2%).

These findings for the Ironman distance partly match with our findings for the Double Iron ultra-triathlon distance. The smaller sex difference in swim split times might be due to higher economy and mechanical efficiency of swimming in women compared to men [2]. The small sex difference in swim split times matches also with recent findings for sex difference in ultra-swim performances in the 26.4-km open-water ultra-swim ‘Marathon Swim in Lake Zurich’. Eichenberger et al. reported a sex difference of 11.5% in open-water ultra-swimming performance in the last 25 years [16]. In the English Channel Swim, no difference in the annual performance between women and men from 1900 to 2010 was found [14]. A possible explanation for these findings could be a reduced surface area in the water giving some specific advantage for female triathletes in swimming performance [25]. In ultra-triathlons, swimming has less influence on the overall race time than running or cycling [26,27]. In a Triple Iron ultra-triathlon, the swim split seemed to have the lowest influence on overall race time [27]. Swimming requires 8.6% of the overall race time in a Triple Iron ultra-triathlon, but cycling and running require 48.5% and 43.6%, respectively [26,27]. The run split seemed to be the most important split discipline for a fast race time in an ultra-triathlon such as a Triple Iron ultra-triathlon [26,27].

### Changes in sex difference over time

Across years, the annual fastest men improved overall race times and split times in running. The annual three fastest men improved overall race times and both split times in cycling and running. Women, however, showed no changes in performance. The sex difference in overall race times and split times remained unchanged over time. Previous studies confirmed this finding and reported a stabilization in sex difference in endurance performance in the early 1980s [13]. The sex differences of overall race time and split times were between 15% and 30% when regarding the fastest athletes ever and the changes in sex difference over time. These values were considerably higher compared to recent reports for Ironman triathletes [3]. A recent study investigating the change in sex difference in performance in Ironman Hawaii showed a decrease in sex difference for overall race time (from 15.2% to 11.3%), an unchanged sex difference in swimming (12.5 ± 3.7%) and cycling (12.5 ± 2.7%), and a decrease in the sex difference in running from 13.5% to 7.3% [3].

A sex gap in endurance performance of 11%–12% seems to be of biological origin. Endurance performance is influenced by aerobic capacity and muscular strength [8]. The higher sex differences in these ultra-endurance athletes might be explained by differences in anthropometric characteristics between female and male ultra-endurance athletes. Anthropometric characteristics such as the body mass index or the distribution of fat and muscle mass in men and women influence ultra-running performance [28]. Body mass index was negatively correlated to average running speed in ultra-marathoners [28]. Men have a higher body mass index than women, which matches with a higher muscle mass in male athletes [28]. A higher body mass index may serve the ultra-marathoners with energy and is a combination of greater fat and muscle mass [28]. A male Ironman triathlete with 41 kg of muscle mass has 31.7% more muscle than a female Ironman triathlete with 28 kg [29]. Percentage of body fat is lower in male triathletes with 13.7% than in female athletes with 23.6% [29]. The greater muscle mass in male ultra-endurance triathletes might be advantageous and might be the reason for faster performance and the sex difference in performance. Female ultra-marathoners, however, might have a greater fatigue resistance than men [30].

### Increase in sex difference with increasing length of endurance performance

The sex differences in both overall race times and split times were considerably higher as reported for Ironman triathletes [1-3]. These differences might be explained by differences in anthropometric characteristics and training between Ironman triathletes and ultra-triathletes [31]. A recent study compared Ironman triathletes and Triple Iron ultra-triathletes, and differences in both anthropometry and training between these two groups of athletes were found [31]. Considering anthropometric characteristics, the Triple Iron ultra-triathletes were smaller, had shorter limbs, a higher body mass index, and larger limb circumferences compared to the Ironman triathletes. Regarding training, the Triple Iron ultra-triathletes invested more hours per week and covered more kilometers. Regarding predictor variables for overall race time, percentage of body fat, weekly training volume, and weekly kilometers in both cycling and running were related to Triple Iron ultra-triathlon race time. For Ironman triathletes, percentage of body fat, circumference of the upper arm, and speed in cycling training were associated with total race time.

A large sex difference in ultra-endurance performance has also been reported for multistage ultra-marathoners competing in the Marathon des Sables [32]. Mean sex difference between 2003 and 2012 was ~31.7% with a decrease from ~39.5% in 2003 to ~24.1% in 2012. In Triple Iron ultra-triathletes, the sex difference in overall performance increased from ~10% in 1992 to ~42% in 2011 for the annual fastest finishers. In cycling and running, the sex differences increased from ~12% to ~40% and from ~10% to ~64%, respectively [19]. Apart from physiological and anthropometric characteristics, the motivation in men with the greater training motivation compared to women [23,24] and the intention to win in male–male physical competition [22] might explain why men compete faster than women in ultra-endurance performances. Additionally, the generally higher participation of men in competitions explains the faster performance in men [23].

### Limitations

In the race where the world record in Double Iron ultra-triathlon for women was achieved in 1994 in Huntsville, USA, the swim split was held downriver. The swim time of 76 min was never corrected for the current in the river, but is still held in the record statistics of the IUTA [33]. Factors such as anthropometry [28,31,34], physiology [35], previous experience [34,36], training [29,31,34,37], pacing strategy [36], motivation [21], nutrition [38,39], nationality [40,41], and equipment [42] may influence overall race time, but were not considered. The influence of environmental factors on the performance of the triathletes was not considered [43-47].

## Conclusions

Men were faster than women in Double Iron ultra-triathlon, men improved in overall race times, cycling and running split times, and the sex difference remained unchanged across years for overall race time and split times. The sex differences for overall race times and split times were higher than reported for Ironman triathlon but lower than reported for Triple Iron ultra-triathlon. Future studies need to investigate the sex difference in performance for longer triathlon distances such as the Quintuple Iron ultra-triathlon and the Deca Iron ultra-triathlon.

## Competing interests

The authors declare that they have no competing interests.

## Authors’ contributions

KS wrote the manuscript. BK and PK collected the data. CAR and RL performed the statistical analyses. TR participated in the design and coordination and helped draft the manuscript. All authors read and approved the final manuscript.

## Funding

The authors received no funding.
